# Management of Fetal Growth Arrest in One of Dichorionic Twins: Three Cases and a Literature Review

**DOI:** 10.1155/2015/289875

**Published:** 2015-12-29

**Authors:** Shoji Kaku, Fuminori Kimura, Takashi Murakami

**Affiliations:** Department of Obstetrics and Gynecology, Shiga University of Medical Science, Shiga 520-2192, Japan

## Abstract

Progressive fetal growth restriction (FGR) is often an indication for delivery. In dichorionic diamniotic (DD) twin pregnancy with growth restriction only affecting one fetus (selective fetal growth restriction: sFGR), the normal twin is also delivered prematurely. There is still not enough evidence about the optimal timing of delivery for DD twins with sFGR in relation to discordance and gestational age. We report three sets of DD twins with sFGR (almost complete growth arrest affecting one fetus for ≥2 weeks) before 30 weeks of gestation. The interval from growth arrest to delivery was 21–24 days and the discordance was 33.7–49.8%. A large-scale study showed no difference of overall mortality or the long-term outcome between immediate and delayed delivery for FGR, while many studies have identified a risk of developmental delay following delivery of the normal growth fetus before 32 weeks. Therefore, delivery of DD twins with sFGR should be delayed if the condition of the sFGR fetus permits in order to increase the gestational age of the normal growth fetus.

## 1. Introduction

When fetal growth restriction (FGR) is progressive, with no increase of the estimated fetal body weight (EFW) and deterioration of Doppler flow parameters measured at the umbilical artery and ductus venosus, delivery is required. However, there is little consensus about the optimal timing of delivery [1]. Early delivery carries the risks associated with prematurity, but delay may increase hypoxic damage [2]. In monochorionic twins, one fetus may show growth restriction while the growth of the other fetus is normal. This is called selective fetal growth restriction (sFGR) and its frequency is 10–15%. However, there have been no reports about the management of dichorionic diamniotic (DD) twin pregnancy with sFGR. Over the past few decades, the incidence of twin pregnancies has increased by nearly 70% because of the widespread use of assisted reproductive technology [3], which means that DD twin pregnancies have also been increasing. Inde et al. reported that 32.9% of patients who had DD twins received in vitro fertilization [4]. Accordingly, we reviewed our cases and the literature to investigate the management and timing of delivery in DD twins with sFGR and almost complete growth restriction.

## 2. Case Reports

We searched the clinical records of our hospital from January 2009 to December 2013 for DD twins with sFGR diagnosed before 30 weeks of gestation. Twins were eligible when the EFW of one twin was below the 10th percentile and there was almost complete growth restriction for more than two weeks, while the EFW of the other twin was within the normal range based on a weight nomogram. We excluded cases where the FGR fetus had an abnormal karyotype. Three twin pregnancies were identified that met these criteria. For these fetuses, we examined the period between the diagnosis of growth restriction and delivery in relation to the prognosis of both twins. In all pregnancies, gestational age was confirmed and chorionicity and amnionicity were evaluated prior to 12 weeks. Gestational age was assigned by measurement of crown-rump length. The EFW of the twin with sIUGR was determined by ultrasound once or twice a week with a Voluson E8 (GE Healthcare, Milwaukee, WI).

For management of sFGR in DD twins, the mother was hospitalized. CTG monitoring was performed every day and the EFW was assessed by ultrasound, with both EFW and Doppler examination being done twice a week. If late deceleration or reduced short-term variation was seen or there was an abnormal UA pulsatility index (more than 2 SD above the normal reference mean) or absence of end-diastolic flow in the UA, we considered delivery if the gestational age was more than 32 weeks. If the gestational age was less than 32 weeks, we increased CTG monitoring to two or three times a day and performed daily ultrasound examination. If late deceleration, absence of short-term variation, or reverse end-diastolic flow (RED) was detected, we considered delivery.

**Figure 1:**
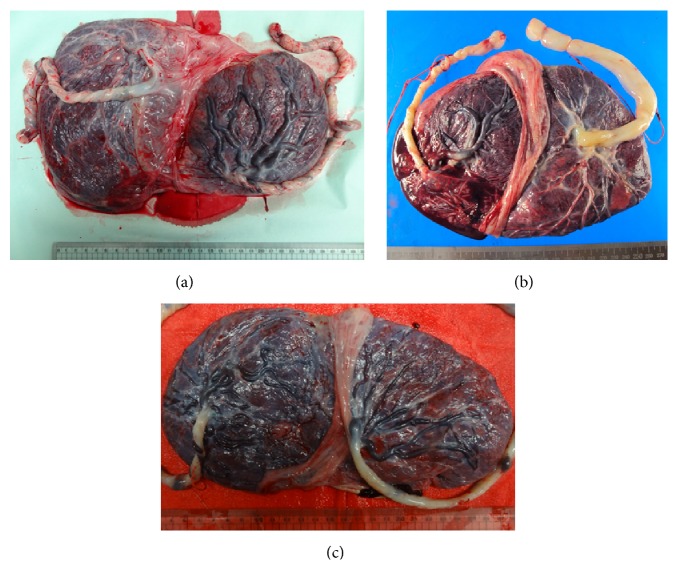
Placentas of 3 cases. (a) Placenta of case 1. (b) Placenta of case 2. (c) Placenta of case 3. (a) There is an obvious difference of placental area between the FGR fetus and the normal fetus. (b, c) There is no marked difference of placental area between the FGR fetus and the normal fetus.

Details of the three cases of sFGR are displayed in Table 1. The gestational age was 27–29 weeks at the detection of almost complete growth restriction persisting for ≥2 weeks, while birth weight discordance was 33.7–49.8% (Table 1). Investigation of the cause of the growth restriction revealed a difference of placental area between the FGR twin and normal twin in case 1 (Figure 1(a)), but there was no significant difference in cases 2 and 3 (Figures 1(b) and 1(c)). In case 2, the FGR fetus showed heterotaxia, but the karyotype was normal. In case 3, the cause of growth restriction was not identified despite prenatal and postnatal investigation. The method of delivery was cesarean section in all three cases. Although we aimed for delivery after 32 weeks of gestation, this was only achieved in case 1. In case 2, RED in the umbilical artery was found at 30 weeks of gestation, and cesarean section was performed three days after the appearance RED. In case 3, labor started at 29 weeks in spite of tocolysis. Accordingly, cesarean section was performed at 29–32 weeks of gestation, and the interval from detection of growth arrest to cesarean section was 21–24 days (median: 22.7 days) (Table 1). The birth weight of the FGR twin was 778–884 g and that of the normal twin was 1174–1760 g (Table 1). After follow-up of the sFGR infants for one to four years since birth, no major abnormalities have been found other than heterotaxia in case 2. Among the normal growth infants, cerebral hemorrhage was detected in the normal weight twin of case 2 at 4 days after birth and this child requires ongoing treatment.

## 3. Discussion

When sFGR occurs in DD twins, our objective is to achieve the best outcome for both fetuses. The timing of delivery is generally the major issue in severe FGR and policies about delivery vary widely [5, 6]. A large-scale prospective study showed that the developmental quotient was significantly lower at a corrected age of 2 years after premature delivery of normal growth fetuses between 22 and 32 weeks of gestation [7]. There is no consensus about the management of sFGR in DD twins, including the timing of delivery. Accordingly, we reviewed published reports on the management of sFGR and investigated the timing of delivery in relation to the severity of discordance to determine whether discordance influenced the normal twin because an adverse event occurred in one of our normal growth twins. We also investigated the timing of delivery in relation to the umbilical artery Doppler flow parameters in the sFGR fetus because RED was found in one of our cases.

With regard to the timing of delivery in relation to the severity of discordance, we found that discordance exceeded 30% in all 3 of our DD twins. In most studies, the cut-off value is 15%–25%, and it is reported that the risk of morbidity and mortality increases if discordance exceeds that value [8–10]. Unfortunately, there have been no reports focusing on the relation between discordance and prognosis of DD twins, but some studies have investigated the influence of gender. In same sex twins, Demissie et al. reported that greater discordance is associated with an increased risk of intrauterine death for both smaller and larger twin, while intrauterine death and the prognosis of the larger twin are unrelated to discordance when the twins are of different sexes [11]. The same sex twins in these reports included both DD twins and monochorionic twins, while the twins of different sexes would only be DD twins. However, the authors did not distinguish between DD twins of the same and DD twins of different sexes, and the chorionicity and amnionicity are also unclear because the studies were based on twin birth data from the United States [11, 12]. However, we considered that the data for different sex twins corresponded to findings for DD twins.

A few prospective multicenter studies have addressed the timing of delivery based on Doppler flow parameters in the umbilical artery of the FGR fetus. The Growth Restriction Intervention Trial (GRIT) investigated the timing of delivery for FGR [13]. Pregnant women between 24 and 36 weeks of gestation with FGR were randomly assigned to immediate delivery (*n* = 296) or delayed (*n* = 291) delivery if the obstetrician was uncertain about when the FGR fetus should be delivered based on UA Doppler parameters. As a result, there was no difference of overall mortality between the two groups. In addition, 2-year and 6-year follow-up studies showed that there were no significant differences between the two groups with regard to death or disability rates [14, 15]. Although the GRIT study cannot provide us with standard criteria for determining the timing of delivery, the lack of a difference in overall mortality and long-term outcomes of the FGR fetus between immediate and delayed delivery suggests that it may be important for parents or obstetricians to consider prolonging the time in utero for the normal twin of DD twins with sFGR, even for a short period.

When we reassessed our 3 cases based on the literature review, we considered that the timing of delivery should not be decided from the discordance (even though it exceeded 30% in all of our cases), because there is no evidence of a relation between the cut-off value for discordance and the risk of morbidity or mortality in dichorionic twins. Although we aimed for delivery after a gestational age of 32 weeks, delivery was earlier than 32 weeks in 2 cases because of RED and spontaneous onset of labor, respectively, with cerebral hemorrhage occurring in one normal growth twin after premature delivery. In conclusion, there is still not enough evidence about the optimal timing of delivery for DD twins with sFGR in relation to discordance and gestational age, but data from the GRIT study suggest that delivery should be delayed if the condition of the sFGR fetus permits in order to increase the gestational age of the normal growth fetus.

## Figures and Tables

**Table 1:** 

Case number	Age	G	P	Gestational age at detection of growth restriction	Gestational age at delivery	Reason for delivery	Period of growth restriction (days)	Birth weight (g)	Sex	Major sequelae
1	38	0	0	29 w 2 d	32 w 4 d	Planning delivery	24	884 1760	F M	— —

2	30	0	0	27 w 0 d	30 w 1 d	RED of UA	23	838 1636	M M	— Cerebral hemorrhage

3	29	0	0	27 w 0 d	29 w 6 d	Onset of labor	21	778 1174	F M	— —

G: gravidity, P: parity, RED: reverse of end-diastolic flow, UA: umbilical artery, F: female, and M: male.
